# The pharmacotherapy of infections in patients with mental disorders receiving psychotropic drugs: Focus on good practices

**DOI:** 10.1192/j.eurpsy.2021.64

**Published:** 2021-08-13

**Authors:** M. Stuhec

**Affiliations:** 1 Clinical Pharmacy, University of Ljubljana, Faculty of Pharmacy & Ormoz Psychiatric Hospital, Ormoz, Slovenia; 2 Clinical Pharmacy & Pharmacology, University of Maribor, Medical Faculty, Maribor, Slovenia

**Keywords:** Infections, Antibiotics, Hospitals and Ambulatory Setting, Psychopharmacology

## Abstract

There is little data on infection treatment in patients with mental disorders, including on the selection of psychotropic, antibiotic, antifungal, and antiviral medications. Bacterial, viral, and fungal infections often occur in patients with mental illnesses, and there is little data on rational pharmacotherapy in this vulnerable population. Antibiotic treatment is a common event during hospitalization in adult psychiatric hospitals and poses a risk of significant potential to almost a quarter of all patients. Most infections are bacterial infections where antibiotics are used, and this topic will be covered in this lecture.

Most patients are being treated for urinary tract infections or respiratory tract infections. The most commonly prescribed antibiotics are co-amoxiclav and cotrimoxazole, followed by ciprofloxacin and nitrofurantoin. Drug-drug interactions (DDIs) between antibiotics and psychotropics often occur, where medications with QTc prolongation potential should be avoided (e.g., some antipsychotics and antidepressants, quinolones, and cotrimoxazole). Penicillins are the most appropriate group, and quinolones should be avoided. DDIs between antibiotics and psychotropic drugs have been reported to occur in 20% of patients, which means that DDIs checking is always necessary before prescribing. Psychiatric adverse events (e.g., hallucinations, restlessness, insomnia) have also been seen in patients with mental disorders.

The participants will learn about general recommendations on antibiotic prescribing in this population, focusing on antibiotics and psychotropics, supported by evidence-based data and real clinical pharmacological tools useful for daily practice.

**Disclosure:**

No significant relationships.

